# Z-Score-Based Modularity for Community Detection in Networks

**DOI:** 10.1371/journal.pone.0147805

**Published:** 2016-01-25

**Authors:** Atsushi Miyauchi, Yasushi Kawase

**Affiliations:** Graduate School of Decision Science and Technology, Tokyo Institute of Technology, Ookayama 2-12-1, Meguro-ku, Tokyo 152-8552, Japan; Université Toulouse 1 Capitole, FRANCE

## Abstract

Identifying community structure in networks is an issue of particular interest in network science. The modularity introduced by Newman and Girvan is the most popular quality function for community detection in networks. In this study, we identify a problem in the concept of modularity and suggest a solution to overcome this problem. Specifically, we obtain a new quality function for community detection. We refer to the function as *Z-modularity* because it measures the Z-score of a given partition with respect to the fraction of the number of edges within communities. Our theoretical analysis shows that Z-modularity mitigates the resolution limit of the original modularity in certain cases. Computational experiments using both artificial networks and well-known real-world networks demonstrate the validity and reliability of the proposed quality function.

## Introduction

Many complex systems can be represented as networks. Analyzing the structure and dynamics of these networks provides meaningful information about the underlying systems. In fact, complex networks have attracted significant attention from diverse fields such as physics, chemistry, biology, and sociology [1, 2].

An issue of particular interest in network science is the identification of community structure [3]. Roughly speaking, a *community* (also referred to as a *module*) is a subset of vertices more densely connected with each other than with nodes in the rest of the network. Note that no absolute definition of a community exists because any such definition typically depends on the specific system at hand. Detecting communities is a powerful way to discover components that have some special roles or possess important functions. For example, consider the network representing the World Wide Web, where vertices correspond to web pages and edges represent the hyperlinks between pages. Communities in this network are likely to be the sets of web pages dealing with the same or similar topics.

There are various methods to detect community structure in networks, which can be roughly divided into two types. First, there are methods based on some conditions that should be satisfied by a community. The most fundamental concept is a clique. A *clique* is a subset of vertices wherein every pair of vertices is connected by an edge. As even a singleton or an edge is a clique, we are usually interested in finding a *maximum clique* or a *maximal clique*, i.e., cliques with maximum size and cliques not contained in any other clique, respectively. Although the definition of a clique is very intuitive, it is too strong and restrictive to use practically. In 2004, Radicchi et al. [4] introduced more practical definitions: a community in a strong sense and a community in a weak sense. A subset *S* of vertices is called a *community in a strong sense* if for every vertex in *S*, the number of neighbors in *S* is strictly greater than the number of neighbors outside *S*. On the other hand, a subset *S* of vertices is called a *community in a weak sense* if the sum, over all vertices in *S*, of the number of neighbors in *S* is strictly greater than the number of cut edges of *S*. Thus, if a subset of vertices is a community in a strong sense, then it is also a community in a weak sense. Recently, Cafieri et al. [5] proposed an enumerative algorithm to list all partitions of the set of vertices into communities in a strong sense with moderate sizes.

Second, but perhaps more importantly, there are methods that maximize a globally defined quality function. The best known and most commonly used quality function is *modularity*, which was introduced by Newman and Girvan [6]. Here let *G* = (*V*,*E*) be an undirected network consisting of *n* = |*V*| vertices and *m* = |*E*| edges. The modularity, a quality function for partition $C=\{C_{1},\ldots ,C_{k}\}$ of *V* (i.e., ${⋃}_{i=1}^{k}C_{i}=V$ and *C*_*i*_ ∩ *C*_*j*_ = ∅ for *i* ≠ *j*), can be written as

$$\begin{array}{c} Q\left(C\right)=\sum_{C\in C}\left(\frac{m_{C}}{m}-{\left(\frac{D_{C}}{2m}\right)}^{2}\right), \end{array}$$

where *m*_*C*_ is the number of edges in community *C*, and *D*_*C*_ is the sum of the degrees of the vertices in community *C*. The modularity represents the sum, over all communities, of the fraction of the number of edges in the communities minus the expected fraction of such edges assuming that they are placed at random with the same distribution of vertex degree.

Many studies have examined modularity maximization. In 2008, Brandes et al. [7] proved that modularity maximization is NP-hard. This implies that unless P = NP, no modularity maximization method that simultaneously satisfies the following exists: (i) finds a partition that maximizes modularity exactly (ii) in time polynomial in *n* and *m* (iii) for any network. To date, a major focus in modularity maximization has been designing accurate and scalable heuristics. In fact, there are a wide variety of algorithms based on greedy techniques [6, 8, 9], simulated annealing [10–12], extremal optimization [13], spectral optimization [14, 15], mathematical programming [16–19], and other techniques. Note that to reduce computation time, a few pre-processing techniques have been proposed [20]. Moreover, to improve the quality of partitions obtained by such heuristics, some post-processing algorithms have also been developed [21].

Although modularity maximization is the most popular and widely used method in practice, it is also known to have some serious drawbacks; i.e., the *resolution limit* [22] and *degeneracies*[23]. The former means that modularity maximization fails to detect communities smaller than a certain scale depending on the total number of edges in a network even if the communities are cliques connected by single edges. The latter means that there exist numerous nearly optimal partitions in terms of modularity maximization, which makes finding communities with maximum modularity extremely difficult. The resolution limit particularly narrows the application range of modularity maximization because most real-world networks consist of communities with very different sizes. To avoid this issue, some multiresolution variants of the modularity have been adopted in practical applications [24–26]. In these variants, the resolution level can be tuned freely by adjusting certain parameters. However, once the resolution level is determined, communities larger than the determined resolution level tend to be divided and smaller communities tend to be merged. Therefore, such multiresolution variants also fail to detect real community structure [27].

In this study, we identify a problem in the concept of modularity and suggest a solution to overcome this problem. Specifically, we obtain a new quality function for community detection. We refer to this function as *Z-modularity* because it measures the Z-score of a given partition with respect to the fraction of the number of edges within communities. Our theoretical analysis shows that Z-modularity mitigates the resolution limit of the original modularity in certain cases. In fact, Z-modularity never merges adjacent cliques in the well-known ring of cliques network with any number and size of cliques. Computational experiments using both artificial networks and well-known real-world networks demonstrate the validity and reliability of the proposed quality function.

Note that there are many other quality functions based on modularity or other concepts [28–33]. Most of them are collected in reference [3].

## Methods

### Definition of Z-modularity

Modularity simply computes the fraction of the number of edges within communities minus its expected value. The definition is quite intuitive; thus, it is the most popular and widely used quality function in practice.

**Fig 1:**
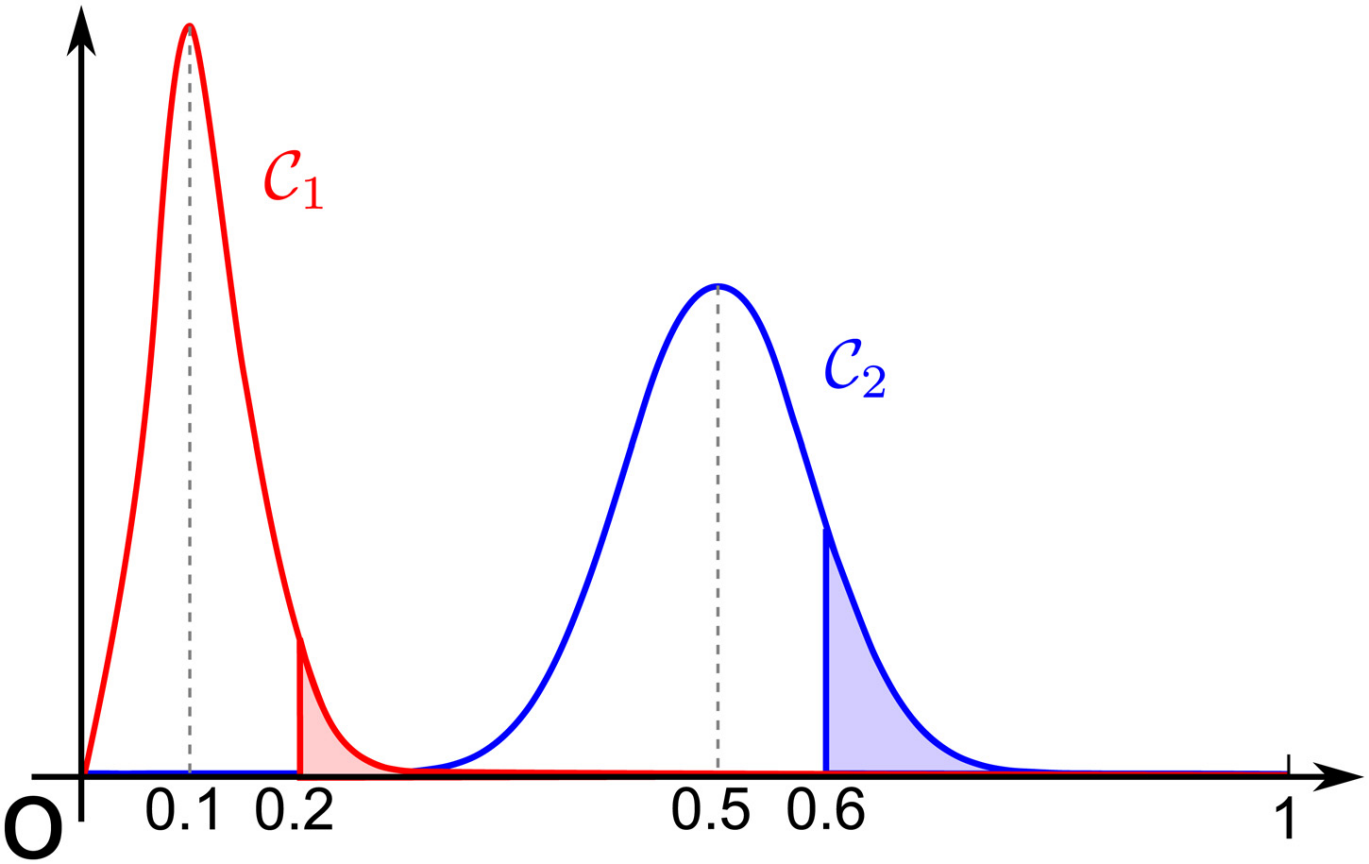
Probability distributions.

However, we identify a problem with the concept of modularity. Here consider two partitions $C_{1}$ and $C_{2}$. Assume that the fraction of the number of edges within communities of $C_{1}$ and $C_{2}$ are 0.2 and 0.6, respectively. In addition, assume that their expected values are 0.1 and 0.5, respectively. Then, we see that these two partitions share the same modularity value (i.e., $Q\left(C_{1}\right)=Q\left(C_{2}\right)=0.1$). The key question is as follows: should these two partitions receive the same quality value? Our answer is that it must depend on the variance of the probability distribution of the fraction of the number of edges within communities of $C_{1}$ and $C_{2}$. Fig 1 illustrates an example. In this case, we wish to assign a higher quality value to $C_{1}$ because it is statistically much rarer than $C_{2}$. This simple but critical observation forms the basis of our quality function.

Given an undirected network *G* = (*V*,*E*) consisting of *n* = |*V*| vertices and *m* = |*E*| edges, and a partition C of *V*, we aim to quantify the statistical rarity of partition C in terms of the fraction of the number of edges within communities. To this end, we consider the following edge generation process over *V*. Place *N* edges over *V* at random with the same distribution of vertex degree. Then, when we place an edge, the probability that the edge is placed within communities is given by

$$\begin{array}{c} p=\sum_{C\in C}{\left(\frac{D_{C}}{2m}\right)}^{2}. \end{array}$$

Note that this edge generation process is the same as the null-model (also known as the *configuration model* [34]) used in the definition of modularity, with the exception of the sample size. We simply wish to estimate the probability distribution of the fraction of the number of edges within communities. Thus, unlike the null-model, the sample size *N* is not necessarily equal to the number of edges *m*.

Let *X* be a random variable denoting the number of edges generated by the process within communities. Then, *X* follows the binomial distribution *B*(*N*,*p*). By the central limit theorem, when the sample size *N* is sufficiently large, the distribution of *X*/*N* can be approximated by the normal distribution N(p,p(1-p)/N). Thus, we can quantify the statistical rarity of partition C in terms of the fraction of the number of edges within communities using the Z-score as follows:

$$\begin{array}{c} Z\left(C\right)=\frac{\sum_{C\in C}\frac{m_{C}}{m}-\sum_{C\in C}{\left(\frac{D_{C}}{2m}\right)}^{2}}{\sqrt{\sum_{C\in C}{\left(\frac{D_{C}}{2m}\right)}^{2}\left(1-\sum_{C\in C}{\left(\frac{D_{C}}{2m}\right)}^{2}\right)}}. \end{array}$$

The sample size *N* never depends on a given partition; thus, it is omitted in the denominator. For partition C with *p* = 0 or 1, we define Z(C)=0. We refer to this quality function as *Z-modularity*.

### Remarks on Z-modularity

The numerator of Z-modularity is none other than modularity. Although it does not make sense to compare the Z-modularity value and the modularity value for a given partition, they have the same sign. In fact, since the denominator of Z-modularity is always positive, Z-modularity takes a positive value if and only if so does modularity. Thus, the positive (resp. negative) value of Z-modularity implies that the fraction of the number of edges within communities is greater (resp. less) than the expected fraction of such edges in the above edge generation process.

Here we provide upper and lower bounds on Z-modularity. As shown in Brandes et al. [7], for any partition C, the modularity value Q(C) falls into the interval [−1/2,1]. On the other hand, Z-modularity has an upper bound of $\sqrt{n}$ and a lower bound of −*n*, as shown below. Recall that $p=\sum_{C\in C}{\left(\frac{D_{C}}{2m}\right)}^{2}$. As for the upper bound, since *p* ≥ 1/*n* (unless *p* = 0), we have

$$\begin{array}{c} Z\left(C\right)=\frac{\sum_{C\in C}\frac{m_{C}}{m}-p}{\sqrt{p(1-p)}}\leq \frac{1-p}{\sqrt{p(1-p)}}=\sqrt{\frac{1}{p}-1}\leq \sqrt{n-1}<\sqrt{n}. \end{array}$$

As for the lower bound, since $p\leq {\left(\frac{2m-1}{2m}\right)}^{2}+{\left(\frac{1}{2m}\right)}^{2}=1-\frac{1}{m}+\frac{1}{2m^{2}}\leq 1-\frac{1}{2m}<1-\frac{1}{n^{2}}$ (unless *p* = 1), we have

$$\begin{array}{c} Z\left(C\right)=\frac{\sum_{C\in C}\frac{m_{C}}{m}-p}{\sqrt{p(1-p)}}\geq -\sqrt{\frac{p}{1-p}}>-n. \end{array}$$

Finally, we mention the computation time concerning Z-modularity. Although Z-modularity is of a more complicated form than modularity, the computation time of the function value is almost the same. On the other hand, if we replace modularity with Z-modularity in some modularity maximization algorithms, their running time may change drastically because, for instance, the number of iterations to converge to a local optimum may increase or decrease substantially. However, as an example, our computational experiments confirmed that the simulated annealing algorithm proposed by Guimerà and Amaral [10] and its Z-modularity maximization version run in almost the same time.

## Theoretical Results

Fortunato and Barthélemy [22] pointed out the resolution limit of modularity. This resolution limit means that modularity maximization fails to detect communities that are smaller than a certain scale depending on the total number of edges in a network. This phenomenon occurs even if the communities are cliques connected by single edges. Here we theoretically analyze Z-modularity from a resolution limit perspective. As a result, we demonstrate that Z-modularity mitigates the resolution limit of the original modularity in certain cases.

### Ring of cliques network

**Fig 2:**
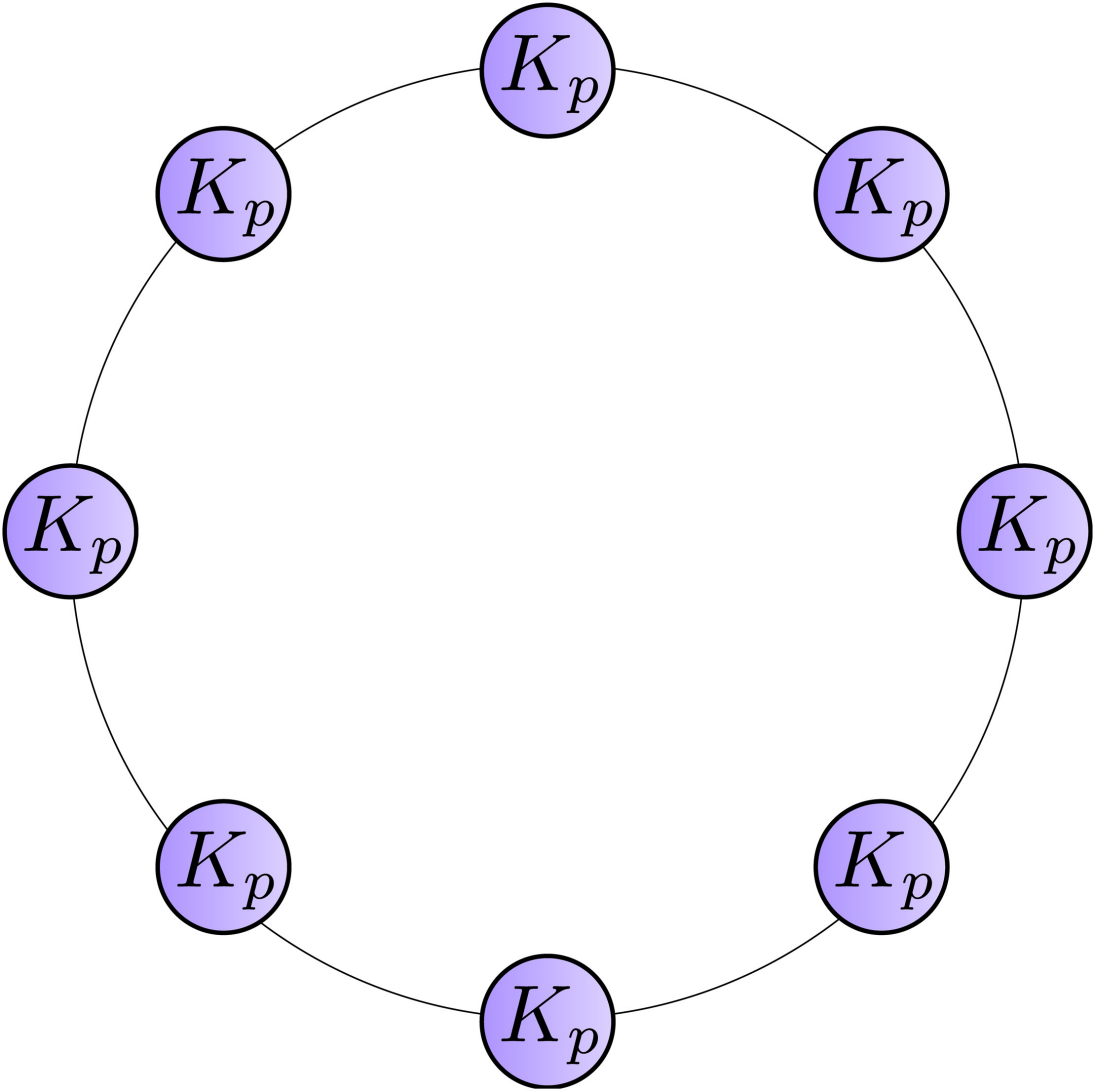
Ring of cliques network. *K*_*p*_ represents a clique with *p* vertices.

First, we consider a *ring of cliques network* that consists of a number of cliques connected by single edges (Fig 2). Assume that each clique consists of *p* (≥3) vertices and the number of cliques is *q* (≥2). Then, the network has *n* = *p*⋅*q* vertices and *m* = *q*⋅(1+*p*(*p*−1)/2) edges. Fortunato and Barthélemy [22] showed that modularity maximization would merge adjacent cliques if *q* is larger than a certain value depending on *p*. However, adjacent cliques are never merged in a partition with maximal Z-modularity value, as shown below.

Let $C^{*}$ be the partition of *V* into the cliques. In addition, let $C=\{C_{1},\ldots ,C_{l}\}$ (1 < *l* < *q*) be a partition of *V* such that each *C*_*i*_ consists of a series of *s*_*i*_ (≥1) cliques and $q=\sum_{i=1}^{l}s_{i}$. Then, Z-modularity for $C^{*}$ and C are calculated by

$$\begin{array}{c} Z\left(C^{*}\right)=\frac{1-q/m-1/q}{\sqrt{(1-1/q)/q}}\;\text{and}\;Z\left(C\right)=\frac{1-l/m-t}{\sqrt{t(1-t)}}, \end{array}$$

respectively, where $t=\sum_{i=1}^{l}{\left(s_{i}/q\right)}^{2}$. By the Cauchy–Schwarz inequality, we have $1>t=\sum_{i=1}^{l}{\left(s_{i}/q\right)}^{2}\geq (\sum_{i=1}^{l}{\left(s_{i}/q\right)}^{2}/l=1/l$. To analyze the behavior of the function Z(C) in details, we rewrite Z(C) using two variables *x* and *y* as follows:

$$\begin{array}{c} f\left(x,y\right)=\frac{1-y/m-x}{\sqrt{x(1-x)}}. \end{array}$$

Then, the derivative of *f*(*x*,*y*) with respect to *x* is

$$\begin{array}{cc} \frac{\partial }{\partial x}f\left(x,y\right) & =\frac{-x\cdot y/m-(1-y/m)(1-x)}{2\cdot {\left(x(1-x)\right)}^{3/2}}<0 \end{array}$$

for 0 < *x* < 1 and 1 ≤ *y* ≤ *m*. Thus, we obtain

$$\begin{array}{c} f(1/l,l)\geq f(t,l). \end{array}$$

Moreover, the derivative of *f*(1/*y*,*y*) with respect to *y* is

$$\begin{array}{cc} \frac{\partial }{\partial y}f\left(1/y,y\right) & =\frac{(m-3y)(y-1)+y}{2m\cdot {\left(y-1\right)}^{3/2}}>0 \end{array}$$

for 1 < *y* < *m*/3. Thus, we have

$$\begin{array}{c} f(1/q,q)>f(1/l,l), \end{array}$$

since 1 < *l* < *q* ≤ *m*/4 by *m* = *q*⋅(1 + *p*(*p*−1)/2) ≥ 4*q*. Therefore, we have

$$\begin{array}{c} Z\left(C^{*}\right)=f\left(1/q,q\right)>f\left(1/l,l\right)\geq f\left(t,l\right)=Z\left(C\right), \end{array}$$

which means that maximizing Z-modularity never merges adjacent cliques.

Table 1 lists the values of modularity and Z-modularity of partitions $C^{*}$ and C (*s*_*i*_ = 2 for *i* = 1, …, *l*) for some ring of cliques networks. As can be seen, the modularity of C is greater than that of $C^{*}$ when the number of cliques is large, which is consistent with Fortunato and Barthélemy [22]. On the other hand, as we proved above, Z-modularity of $C^{*}$ is certainly higher than that of C for every number of cliques.

**Table 1:** Numerical examples of modularity and Z-modularity for some ring of cliques networks.

*n*	*m*	*p*	*q*	$Q(C^{*})$%	Q(C)%	$Z(C^{*})$%	Z(C)%
100	220	5	20	**0.8591**	0.8548	**3.942**	2.848
200	440	5	40	0.8841	**0.9045**	**5.663**	4.150
400	880	5	80	0.8966	**0.9295**	**8.070**	5.954
5000	11000	5	1000	0.9081	**0.9525**	**28.73**	21.32

### Network with two pairwise identical cliques

**Fig 3:**
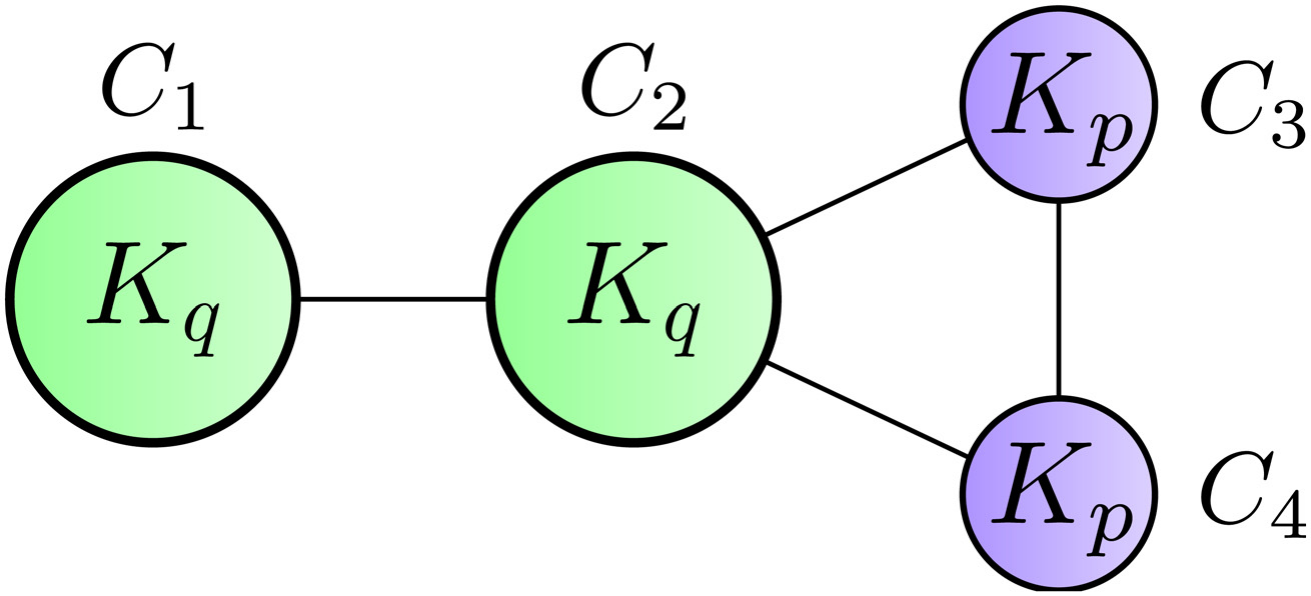
Network with two pairwise identical cliques. *K*_*p*_ and *K*_*q*_ represent cliques with *p* and *q* vertices, respectively.

Here we consider a *network with two pairwise identical cliques* that consists of a pair of cliques *C*_1_ and *C*_2_ with *q* vertices each and a pair of cliques *C*_3_ and *C*_4_ with *p* (< *q*) vertices each. These four cliques are connected by single edges, as described in Fig 3. This network has *n* = 2(*p* + *q*) vertices and *m* = *p*(*p*−1) + *q*(*q*−1) + 4 edges.

Consider two partitions $C_{A}=\left\{C_{1},C_{2},C_{3},C_{4}\right\}$ and $C_{B}=\left\{C_{1},C_{2},C_{3}\cup C_{4}\right\}$. Note that partition $C_{A}$ is a more natural community structure that we would like to identify. Unfortunately, maximizing Z-modularity may choose $C_{B}$, i.e., $Z\left(C_{A}\right)<Z\left(C_{B}\right)$ holds for some pair of *p* and *q*. However, if modularity maximization adopts $C_{A}$, then so does Z-modularity, i.e., for any pair of *p* and *q*, if $Q\left(C_{A}\right)>Q\left(C_{B}\right)$ holds, then $Z\left(C_{A}\right)>Z\left(C_{B}\right)$ also holds. This fact follows directly from the definitions of Z-modularity and the original modularity.

Table 2 lists the values of modularity and Z-modularity of partitions $C_{A}$ and $C_{B}$ for some networks with two pairwise identical cliques. We can confirm that both modularity and Z-modularity tend to merge *C*_3_ and *C*_4_ as the sizes of *C*_1_ and *C*_2_ become large. However, there is the case where only Z-modularity could divide *C*_3_ and *C*_4_. Therefore, we see that Z-modularity again mitigates the resolution limit of modularity in this case.

**Table 2:** Numerical examples of modularity and Z-modularity for some networks with two pairwise identical cliques.

*n*	*m*	*p*	*q*	$Q(C_{A})$	$Q(C_{B})$	$Z(C_{A})$	$Z(C_{B})$
26	80	5	8	**0.6618**	0.3385	**1.443**	1.345
42	264	5	16	0.5650	**0.5653**	**1.144**	1.143
74	1016	5	32	0.5182	**0.5190**	1.037	**1.039**
138	4056	5	64	0.5047	**0.5049**	1.009	**1.010**

## Experimental Results and Discussion

The purpose of our computational experiments is to evaluate the validity and reliability of the quality function Z-modularity. To this end, throughout the experiments, we maximize Z-modularity using a simulated annealing algorithm. Note that our algorithm is obtained immediately by changing the objective function from modularity to Z-modularity in the algorithm proposed by Guimerà and Amaral [10]. The implementation of their algorithm can be found on Lancichinetti’s web page [35], and we use it with default parameters with the exception of the above change of objective function. Our experiments are conducted on various artificial networks and on well-known real-world networks.

### Artificial networks

First, we report the results of computational experiments with artificial networks. We compare partitions obtained by maximizing Z-modularity with partitions obtained by modularity maximization on a wide variety of networks. The modularity is also maximized by the simulated annealing algorithm proposed by Guimerà and Amaral [10]. We deal with three types of artificial networks: the planted *l*-partition model, the Lancichinetti–Fortunato–Radicchi (LFR) benchmark, and the Hanoi graph. For the planted *l*-partition model and the LFR benchmark, once their parameters are set, the ground-truth community structure is fixed. Thus, we can evaluate the quality of the obtained community structure by comparison with the ground-truth using some measure.

To this end, we adopt the *normalized mutual information* for two partitions, which was introduced by Fred and Jain [36]. The normalized mutual information for two partitions $C_{1}$ and $C_{2}$ of *n* vertices is defined as follows:

$$\begin{array}{c} I_{\text{norm}}\left(C_{1},C_{2}\right)=\frac{2I(C_{1},C_{2})}{H\left(C_{1}\right)+H\left(C_{2}\right)}, \end{array}$$

where

$$\begin{array}{c} I\left(C_{1},C_{2}\right)=\sum_{C_{1}\in C_{1}}\sum_{C_{2}\in C_{2}}\frac{|C_{1}\cap C_{2}|}{n}\log_{2}\left(\frac{n\cdot |C_{1}\cap C_{2}|}{|C_{1}\left|\cdot \right|C_{2}|}\right) \end{array}$$

and

$$\begin{array}{c} H\left(C\right)=-\sum_{C\in C}\frac{\left|C\right|}{n}\log_{2}\frac{\left|C\right|}{n}. \end{array}$$

The normalized mutual information ranges from 0 to 1. For two partitions $C_{1}$ and $C_{2}$, the higher the normalized mutual information is, the more similar they are (and vice versa). In fact, $I_{\text{norm}}\left(C_{1},C_{2}\right)=1$ if $C_{1}$ and $C_{2}$ are identical, and $I_{\text{norm}}\left(C_{1},C_{2}\right)=0$ if they are independent. This measure has often been used to evaluate community detection methods. For example, see the computational experiments in references [37, 38].

#### Planted *l*-partition model

The planted *l*-partition model was introduced by Condon and Karp [39]. In this model, *n* vertices are divided into *l* equally sized groups (with size *c* = *n*/*l*). Two vertices in the same group are connected by probability *p*_in_, whereas two vertices in different groups are connected by probability *p*_out_ (< *p*_in_). Throughout the experiments, we set *p*_in_ = 0.5. We construct four networks corresponding to combinations of two different network sizes (*n* = 1000 or 5000) and two different community sizes (*c* = 20 or 50). The parameter *p*_out_ starts with 0.01 and then increases in stages.

**Fig 4:**
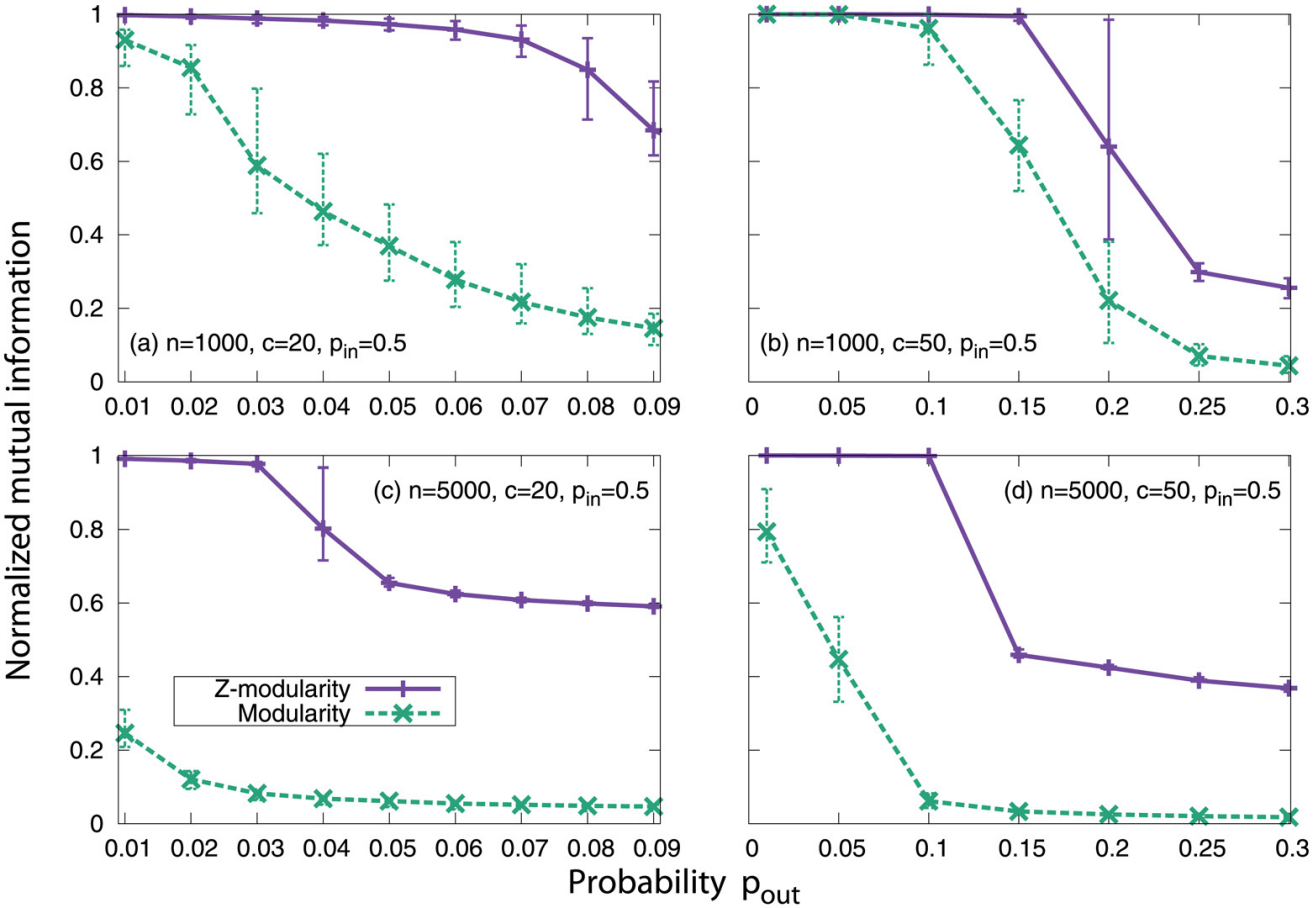
Results for the planted *l*-partition model. Each point is the result of averaging over 100 network realizations. The top and bottom bars represent the maximum and minimum values, respectively.

The results are shown in Fig 4. As can be seen, Z-modularity outperforms the original modularity in all four cases. In particular, Z-modularity provides much more superior results compared to modularity for networks consisting of relatively small communities.

#### LFR benchmark

In the planted *l*-partition model, each group in a generated network forms the Erdő–Rényi random graph [40]. Thus, all vertices have approximately the same degree. Moreover, all groups have exactly the same size. These phenomena are rarely observed in networks in real-world systems. As a more realistic model, the LFR benchmark was proposed by Lancichinetti, Fortunato, and Radicchi [41] for the case of unweighted and undirected networks. The LFR benchmark was then extended to the case of directed and weighted networks with overlapping communities [42]. We now use the original unweighted and undirected case without overlapping communities.

In the model, degree distribution and community size distribution follow power laws with exponents *γ* and *β*, respectively. Furthermore, we can specify the number of vertices *n*, average degree 〈*k*〉, maximum degree *k*_max_, minimum community size *c*_min_, maximum community size *c*_max_, and mixing parameter *μ*. In particular, mixing parameter *μ* indicates the mixing ratio of communities, i.e., the higher *μ* is, the more densely connected the communities are. The model constructs a network consistent with the specified parameters. For more details, see reference [41]. In our experiments, we set the parameters as in references [37, 38] as follows: *γ* = −2, *β* = −1, 〈*k*〉 = 20, and *k*_max_ = 50. We construct eight networks corresponding to combinations of two different network sizes (*n* = 1000 or 5000) and four different ranges of community size ((*c*_min_, *c*_max_) = (10,50), (20,100), (30,150), or (40,200)).

**Fig 5:**
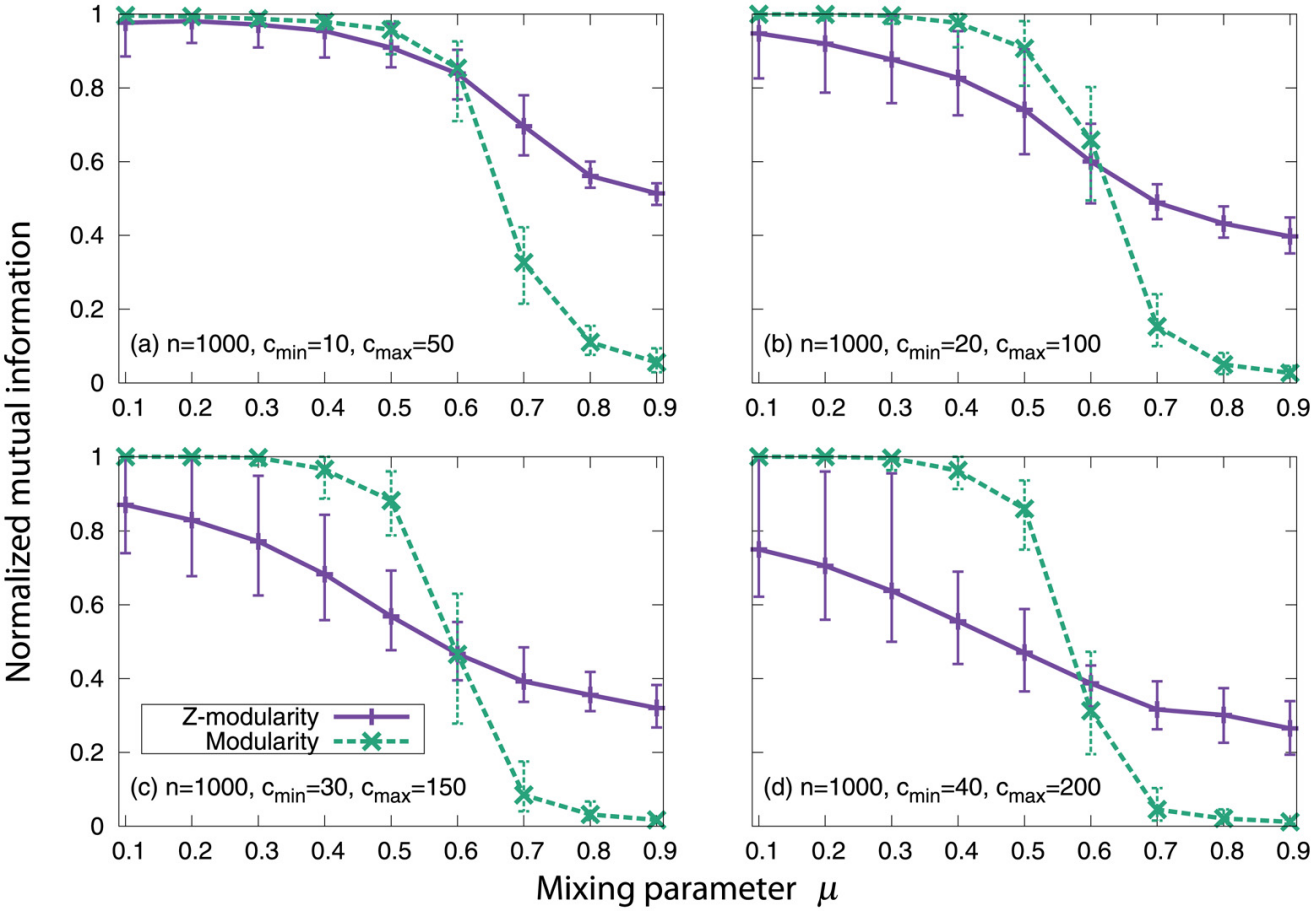
Results for the LFR benchmark (*n* = 1000). Each point is the result of averaging over 100 network realizations. The top and bottom bars represent the maximum and minimum values, respectively.

**Fig 6:**
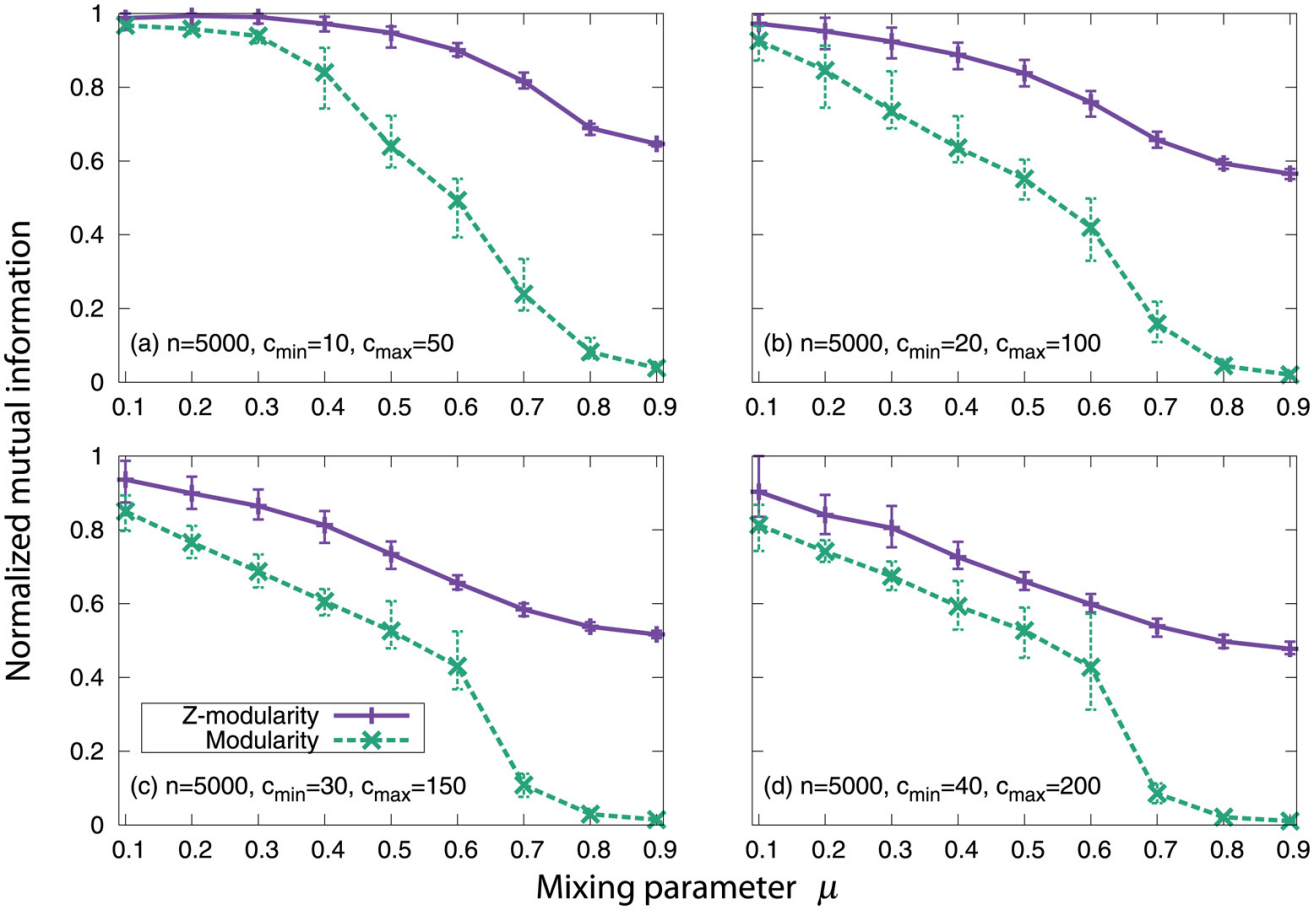
Results for the LFR benchmark (*n* = 5000). Each point is the result of averaging over 100 network realizations. The top and bottom bars represent the maximum and minimum values, respectively.

The results are illustrated in Fig 5 (*n* = 1000) and Fig 6 (*n* = 5000). For the smaller networks (*n* = 1000), the mutual information values obtained by maximizing Z-modularity are lower than those obtained by modularity maximization when *μ* ≤ 0.5 for all community size settings. This trend is significant when the network consists of relatively large communities (e.g., (*c*_min_, *c*_max_) = (30,150) and (40,200)). On the other hand, for larger networks (*n* = 5000), Z-modularity outperforms the original modularity for all community size settings. From the above, we see that Z-modularity is particularly suitable for identifying community structure when a network consists of relatively small communities.

**Fig 7:**
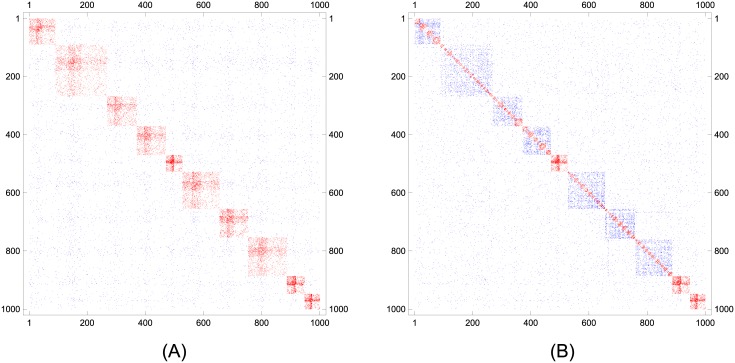
Adjacency matrices for an LFR benchmark network. (A) Ground-truth partition: 10 communities. (B) Optimal partition for Z-modularity: 81 communities and *I*_norm_ = 0.6942.

Here we investigate why the mutual information values obtained by maximizing Z-modularity are low when the community sizes are large. To this end, Fig 7 depicts the adjacency matrices of the LFR benchmark network with parameters *γ* = −2, *β* = −1, *n* = 1000, 〈*k*〉 = 20, *k*_max_ = 50, *c*_min_ = 40, *c*_max_ = 200, and *μ* = 0.3. The vertices are ordered according to both the ground-truth partition and the optimal partition for Z-modularity. The edges connecting vertices in the same community and in different communities are plotted with different colors, i.e., red and blue, respectively. As can be seen, maximizing Z-modularity divides the relatively large ground-truth communities because they contain much denser communities in the hierarchical structure by random behavior.

#### Hanoi graph

Here we demonstrate optimal partitions with respect to Z-modularity and the original modularity for the Hanoi graph, which is an example of networks with hierarchical organization. The Hanoi graph *H*_*n*_ corresponds to the allowed moves in the *tower of Hanoi* for *n* disks, which is a famous puzzle invented by Édouard Lucas in 1883. The Hanoi graph *H*_*n*_ has 3^n^ vertices and 3⋅(3^*n*^−1)/2 edges. In the context of community detection in networks, Hanoi graph *H*_3_ is used by Rosvall and Bergstrom [43]. Note that since the Hanoi graph does not have the ground-truth community structure, it is impossible to conclude whether the obtained partition is reasonable; we use this instance to observe the behavior of Z-modularity maximization.

**Fig 8:**
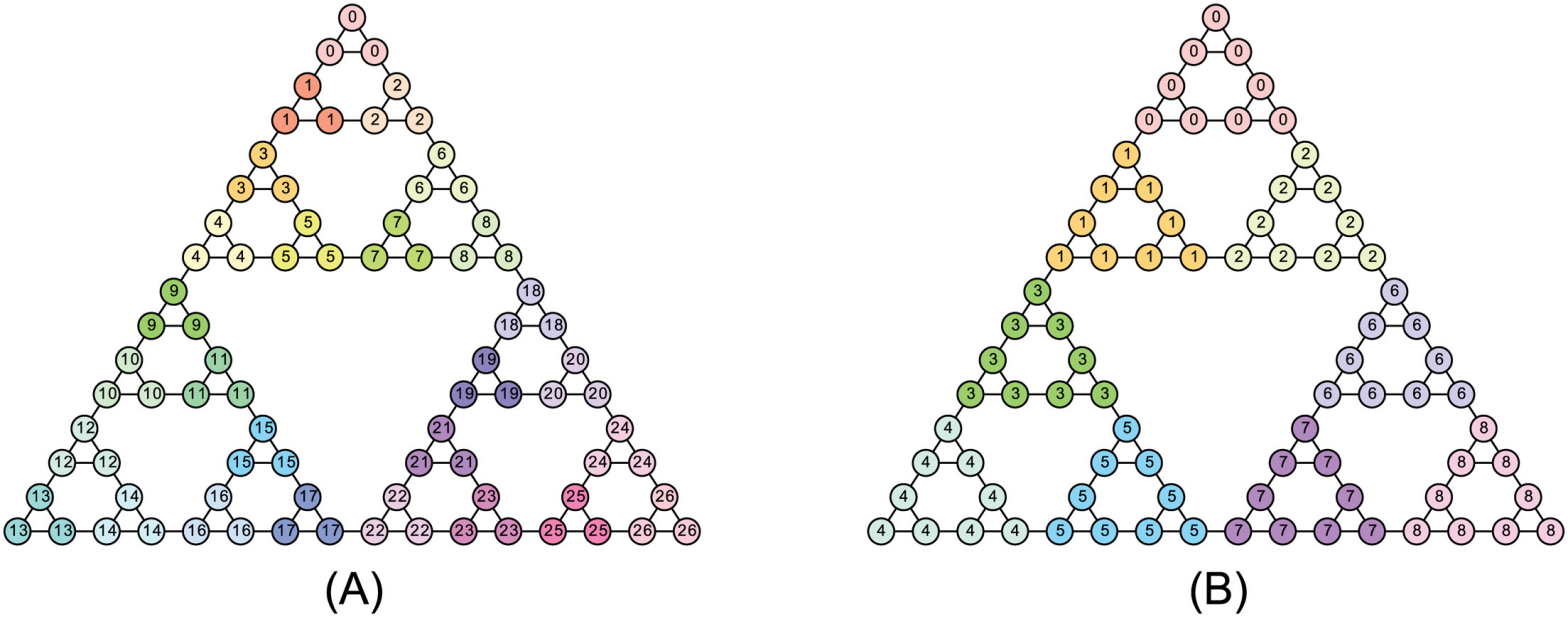
Community structure for Hanoi graph *H*_4_. (A) Optimal partition for Z-modularity: 27 communities, *Z* = 3.376, and *Q* = 0.6379. (B) Optimal partition for modularity: 9 communities, *Z* = 2.510, and *Q* = 0.7889.

The results for Hanoi graph *H*_4_ are shown in Fig 8, where the label (and color) of each vertex represents the community to which the vertex belongs. As can be seen, maximizing Z-modularity leads to more detailed partition than modularity maximization. This result supports the trend observed in the experiments for the planted *l*-partition model and the LFR benchmark.

### Real-world networks

Here we report the results of computational experiments with real-world networks; i.e., the Zachary’s karate club network, the Les Misérables network, and the American college football network.

#### Zachary’s karate club network

The first example is the famous karate club network analyzed by Zachary [44], which is often used as a benchmark to evaluate community detection methods. It consists of 34 vertices representing the members of a karate club in an American university, in addition to 78 edges representing friendship relations among individuals. Because of a conflict between the club administrator and the instructor, the club members split into two groups, one supporting the administrator and the other supporting the instructor. Therefore, these groups can be viewed as a ground-truth community structure.

**Fig 9:**
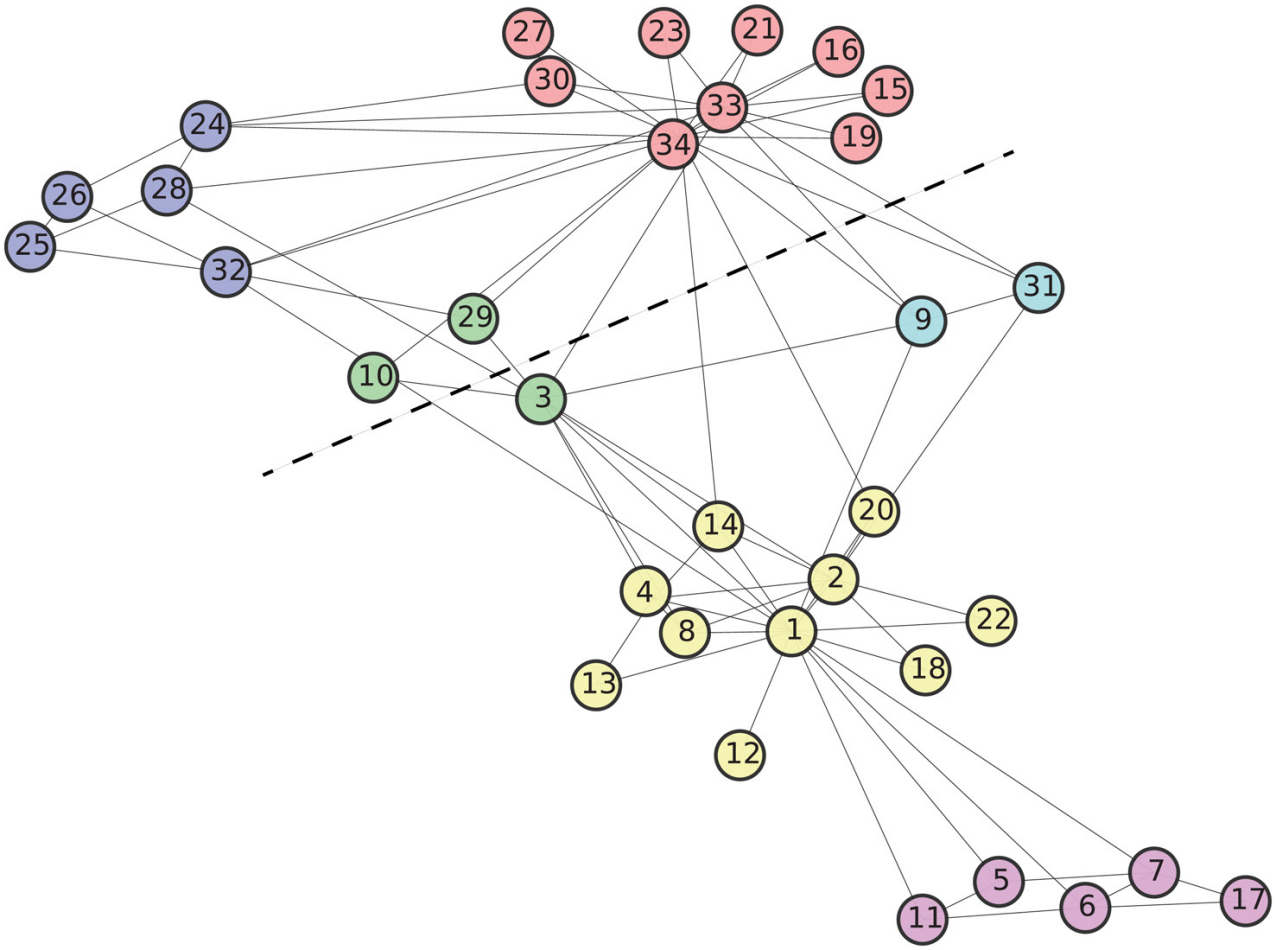
Community structure for Zachary’s karate club network: 6 communities, *Z* = 0.9266, *Q* = 0.3882, and *I*_*norm*_ = 0.4796.

The partition obtained by maximizing Z-modularity is shown in Fig 9, where vertices with the same color represent a community. The label of each vertex represents an identification number of the member. For example, 1 and 34 represent the administrator and the instructor, respectively. The dashed line gives the partition of the network into the above two groups. The mutual information value of the obtained partition is not high. In fact, in comparison with the ground-truth community structure, the obtained partition consists of relatively small communities. This result is consistent with the trend observed in the experiments for artificial networks.

#### Les Misérables network

The second example is the network of the characters in the novel *Les Misérables* by Victor Hugo, compiled by Knuth [45]. It consists of 77 vertices representing the characters and 254 edges indicating the co-appearance of characters. Note here that since this network does not have the ground-truth community structure, it is impossible to evaluate the obtained partition using the mutual information value; we use this network to observe the behavior of Z-modularity maximization.

**Fig 10:**
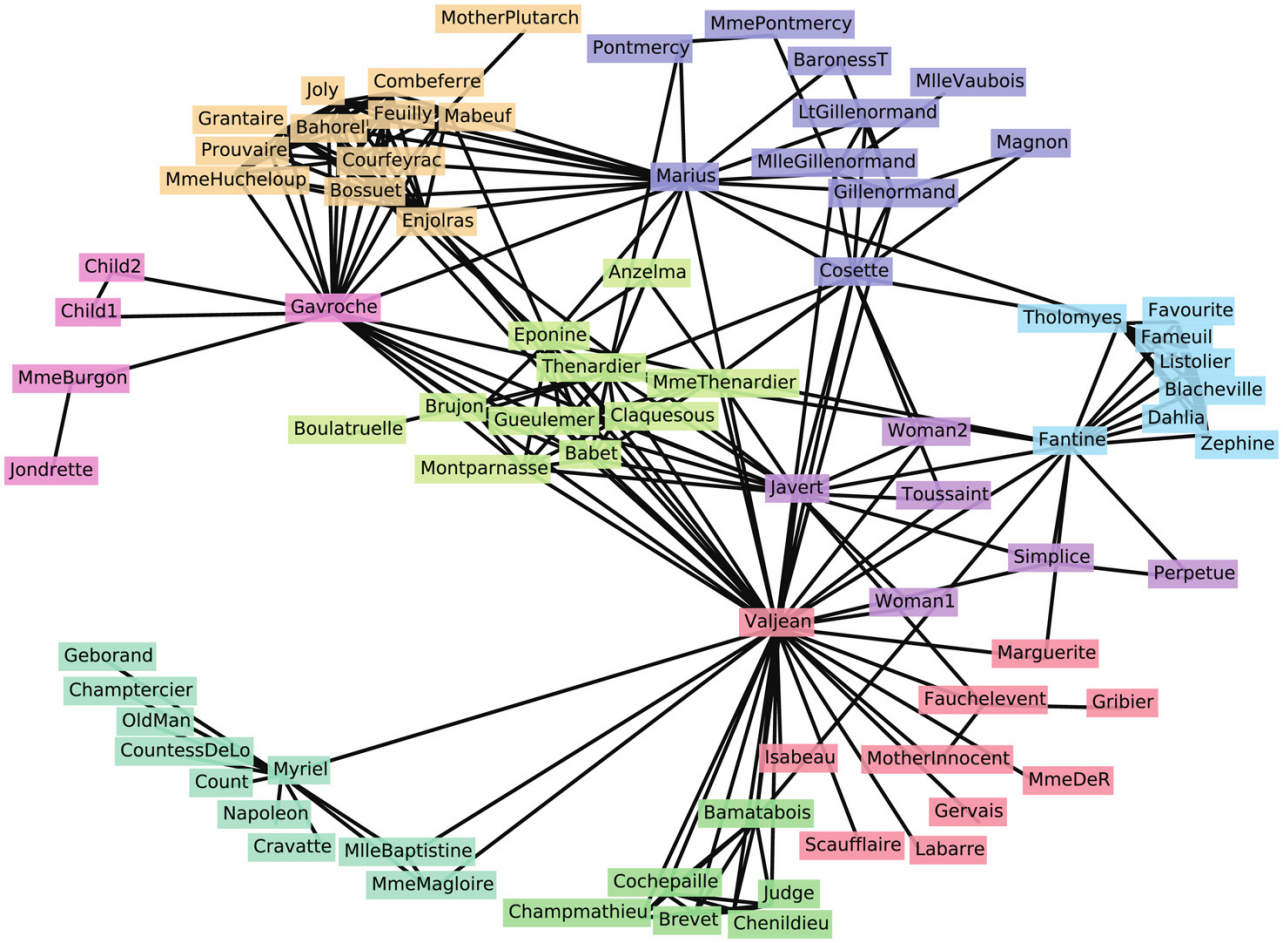
Community structure for Les Misérables network: 9 communities, *Z* = 1.490, and *Q* = 0.5245.

The partition obtained by maximizing Z-modularity is presented in Fig 10, where vertices with the same color represent a community. The label of each vertex represents the name of the character. Identified communities are likely to correspond to specific groups within the story. For example, the community consisting of 12 vertices (shaded with light brown) at the top left corner contains major characters belonging to the revolutionary student club *Friends of the ABC*.

#### American college football network

The third and final example is a network of college football teams in the United States, which was derived by Girvan and Newman [46]. There are 115 vertices representing the football teams, and 654 edges connecting teams that played each other in a regular season. The teams are divided into 12 groups referred to as *conferences* containing approximately 10 teams each. More games are played between teams in the same conference than between teams in different conferences. Thus, the conferences can be viewed as a ground-truth community structure.

**Fig 11:**
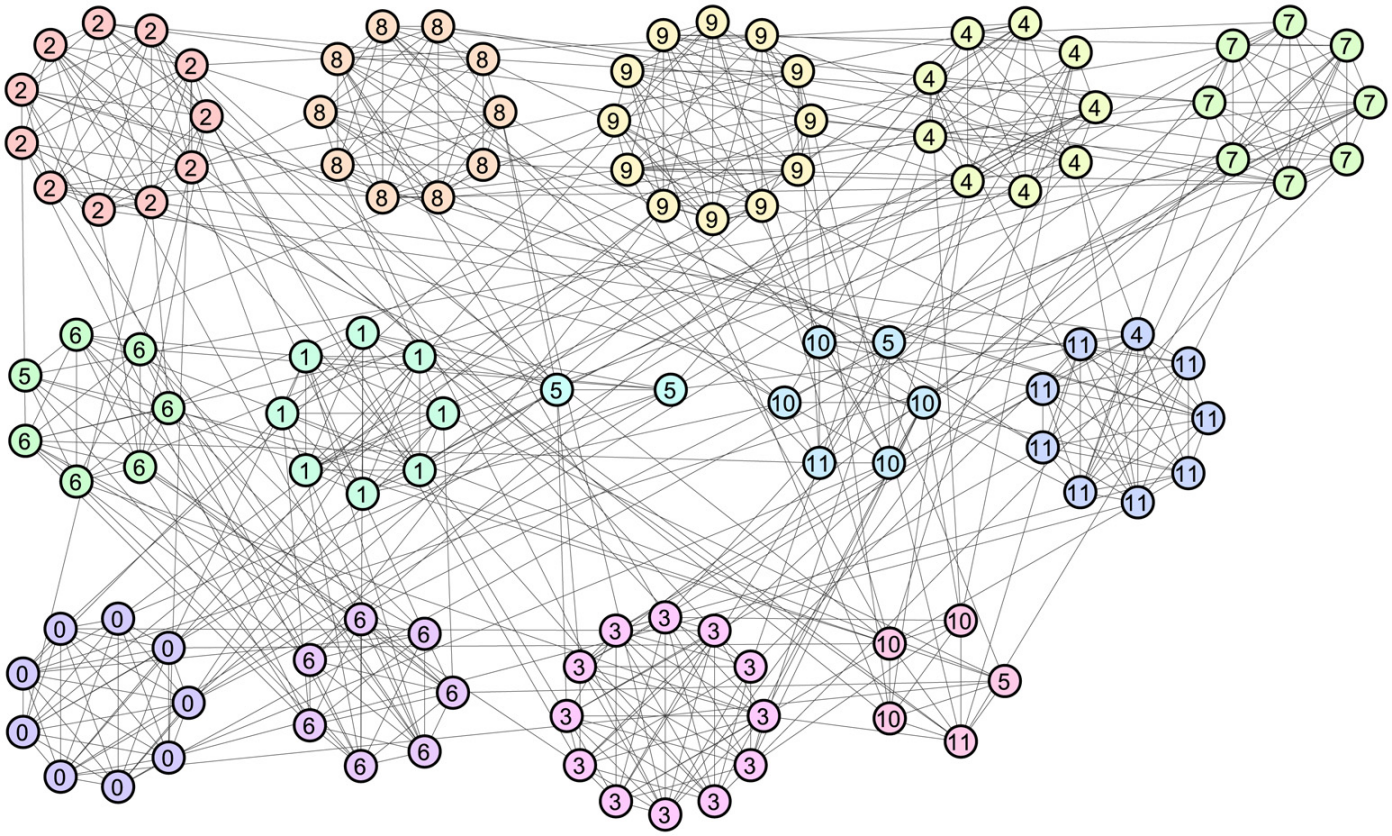
Community structure for American college football network: 14 communities, *Z* = 2.111, *Q* = 0.5738, and *I*_*norm*_ = 0.9205.

The partition obtained by maximizing Z-modularity is shown in Fig 11, where vertices with the same color represent a community. Note that the label of each vertex now represents the conference to which the team belongs rather than an identification number of the team. Although some misclassifications are observed, Z-modularity correctly identifies 7 out of 12 conferences (i.e., conferences 0, 1, 2, 3, 7, 8, and 9). This result is outstanding in comparison with partitions obtained by modularity maximization. In fact, as reported in reference [16], only four conferences were correctly recovered by partition with a higher modularity value *Q* = 0.6046.

## Conclusions

In this study, we have identified a problem in the concept of modularity and suggested a solution to overcome this problem. Specifically, we have obtained a new quality function Z-modularity that measures the Z-score of a given partition with respect to the fraction of the number of edges within communities. Theoretical analysis has shown that Z-modularity mitigates the resolution limit of the original modularity in certain cases. In fact, Z-modularity never merges adjacent cliques in the well-known ring of cliques network with any number and size of cliques. In computational experiments, we have evaluated the validity and reliability of Z-modularity. The results for artificial networks show that Z-modularity more accurately detects the ground-truth community structure than the original modularity in most cases. In particular, Z-modularity outperforms modularity for networks consisting of relatively small communities. Furthermore, the results for real-world networks demonstrate that Z-modularity leads to natural and reasonable community structure in practical use. Therefore, we conclude that Z-modularity could be another option for the quality function in community detection.

In the future, further experiments should be conducted to examine the performance of Z-modularity in more details. In fact, computational experiments in the present study have not used large-scale networks (with heterogeneous community sizes). This is due to the time complexity of the simulated annealing algorithm proposed by Guimerà and Amaral [10], on which our algorithm is based. To conduct computational experiments on large-scale networks, scalable Z-modularity maximization algorithms should be developed. For modularity maximization, there exist a wide variety of fast algorithms that perform well in practice. For example, the greedy algorithm proposed by Blondel et al. [8], which is known as the *Louvain method*, runs in time approximately linear in the size of the network. It should be noted that the corresponding Z-modularity maximization algorithm is derived directly by changing the objective function from modularity to Z-modularity. However, our preliminary experiments demonstrated that the Louvain method is not suitable for Z-modularity maximization. Indeed, the first aggregation step in the algorithm is not effective due to the term in the denominator of Z-modularity.

As another future direction, the physical interpretation of maximizing Z-modularity should be investigated. For example, it is known that modularity maximization can be interpreted as the problem of finding the ground state of a spin glass model [24].
